# Integrating rural and Indigenous culture into pharmacy education: experiential education, preceptor development and Indigenous ways of knowing

**DOI:** 10.3389/fmed.2026.1954823

**Published:** 2026-09-07

**Authors:** Ronny A. Bell, Lisa Kremer, Jaime Tutbury, Jacqueline E. McLaughlin

**Affiliations:** 1 Division of Pharmaceutical Outcomes and Policy, Eshelman School of Pharmacy, University of North Carolina at Chapel Hill, Chapel Hill, NC, United States; 2 He Rau Kawakawa (School of Pharmacy and Pharmacology), Ōtākou Whakaihu Waka (University of Otago), Dunedin, New Zealand; 3 Division of Practice Advancement and Clinical Education, Eshelman School of Pharmacy, University of North Carolina at Chapel Hill, Chapel Hill, NC, United States; 4 Center for Innovative Pharmacy Education & Research, Eshelman School of Pharmacy, University of North Carolina at Chapel Hill, Chapel Hill, NC, United States

**Keywords:** culture, experiential education, Indigenous populations, pharmacy preceptors, rural populations

## Introduction

Health professions programs, including pharmacy, rely extensively on immersive practice experiences integrated with classroom didactic training to ensure that students develop the appropriate attitude, knowledge and skills to be effective health practitioners upon graduation. These immersive experiences are overseen by a preceptor who is a qualified health professional in the clinical or community setting who works with the academic program to provide a structured, hands-on opportunity for the student during a set period of time during their academic training. Preceptors can be highly influential in the professional development of students, and, in some circumstances, become the first employer of these students after finishing their training (1–3). The setting in which these immersive experiences occur can provide important opportunities for cultural and professional development for the student, especially in rural and Indigenous communities.

### Positionality statement

The authorship of this work comprises of three Indigenous authors from two parts of the world (Dr. Ronny Bell, Lumbee Tribe of North Carolina, USA; Dr. Lisa Kremer, Kāi Tahu, Kāti Māmoe, Waitaha of New Zealand; and Ms. Jaime Tutbury, Te Whakatōhea of New Zealand) and a non-Indigenous author (Dr. Jacqui McLaughlin). The authors represent multiple gender identities and educational backgrounds. Although there is diversity in the individual positionality within the authorship, the group has shared positionality as pharmacy educators about the importance of CI in healthcare practice, and the belief that colonization and racism impacts negatively on Indigenous health outcomes.

### Cultural intelligence in experiential education

In many circumstances, the settings for immersive experiences are in communities that are a great distance from, and structurally and culturally different from, that of the academic institution. This experience provides students with a unique opportunity to directly engage with a community to understand the relationship between culture and understanding of health and health care in these communities. From our experience, when students are exposed to, have direct interaction with, and incorporate aspects of culture (e.g., awareness, competency, humility and safety) through informed mentoring and support from preceptors who are established and engaged within their community, students experience a deeper understanding of the community in which they are placed. Importantly, students are able to bring clinical and cultural knowledge gained from the classroom, and be able to experience the application of shared decisions whereby they see the acknowledgement and respect of rural and Indigenous communities as knowledge holders. This reciprocal knowledge exchange experience positions rural and Indigenous communities as educational partners and co-creators of experiential learning. For Indigenous students, being able to bring their own lived experiences into practice and having the opportunity to return home and give back to their community are important cultural values to enact.

This adaptation is referred to as cultural intelligence (CI), defined as the “capability of an individual to effectively function in situations characterized by cultural diversity (4).”

The awareness of the impact CI can have on health is increasing, and because of this, student learning opportunities are being incorporated more into pharmacy pedagogy. A recent systematic review by Ghosh et al., (2025) showed that PharmD programs incorporate CI into their curriculum through reflections, local interests, and regional demographics (5). From a preceptor perspective, results from a recent systematic review conducted by Reti et al., (2026) showed that pharmacist preceptors do not feel well prepared to impart cultural concepts to their trainees, impacting their confidence in fulfilling this responsibility (6). Thus, pharmacy students may get minimal exposure to CI from their preceptors during their experiential learning, potentially impacting their ability to deliver cultural concepts in pharmacist practice. Despite the current ability in preceptor preparedness to adequately develop individual cultural intelligence, it is our view that this learning is essential for students to move into deeper understanding of power, privilege, coloniality and ultimately become health advocates of structural changes required to ensure rural and Indigenous communities are benefiting from the health system.

## Discussion

Health professional students often participate in immersive practice experiences in rural communities, which provides these students an opportunity to understand the unique cultural nuances in these communities (7, 8). Approximately 14% of the US population (9) and 42% of the population around the world (10) live in rural areas. Rural communities experience significant health inequities which are often exacerbated by adverse social determinants of health (e.g., economic instability, inadequate access to health care and quality education, neighborhood and environmental challenges) (11–13) and therefore require equitable approaches to healthcare services and delivery to achieve health gains. Furthermore, students placed in rural settings for experiential learning often experience “placement poverty” through associated travel costs, establishment of temporary residence, and loss of wages, which presents a challenge in encouraging students to select experiential opportunities in these settings (14–16).

Rural communities are not homogenous; many rural communities are racially, ethnically and culturally diverse. The traditional homelands of many of the Indigenous peoples across the world are often located in rural, less population dense areas (17). The intersection of rurality and Indigeneity presents unique challenges for providing health care for these communities, including elements of racism and colonization, lack of transportation options for patients to health care facilities, language and communication barriers, a lack of trust of health care providers and a limited understanding by providers of cultural traditional medical practices. This also limits opportunities for health professional students to gain CI from an Indigenous perspective.

Cultural traditions and educational structures that emphasize objectivity, individualism, and rigid hierarchies often marginalize Indigenous knowledge systems and relational approaches to health (18, 19). Integrating CI in curricula helps learners and preceptors more effectively recognize, value, act upon, and adapt to diverse cultural contexts—fostering humility, curiosity and responsiveness rather than compliance with narrow definitions of “professionalism” (4). Additionally, having a curriculum that has social accountability as a model of practice integrates the education provider as part of the community (20). Social advocacy within curriculum facilities learning on required changes within the education health system and learning activities enable students to identify and advocate for rural and Indigenous health equity and importantly does not place expectations on rural and Indigenous communities to change (21).

For the various cultures embedded within rural communities to be visible in experiential learning, development of reciprocal relationships with a diverse range of rural communities is needed to facilitate effective immersive experiences. Therefore, reciprocal relationships between the preceptor and the academic institution need to be built and maintained over time. By focusing on preceptor development to ensure CI is included in the student immersive experience, and long-term, supportive relationships with diverse rural community partners, this commentary underscores the importance of practice-based educators in disrupting these norms through evidence-based strategies, such as reflective mentorship, relational accountability, and culturally responsive teaching. Implementing this approach across health professions can broaden the opportunities trainees have to become culturally aware and responsive and bridge the gap in health care delivery in these communities to contribute to addressing health inequities.
